# Microbial-Mediated Soil Nutrient Enhancement in Moso Bamboo–*Liquidambar formosana* vs. *Phoebe chekiangensis* Mixed Plantings

**DOI:** 10.3390/plants14121868

**Published:** 2025-06-18

**Authors:** Anming Zhu, Lili Fan, Gang Lu, Liangjin Yao, Jianzhong Fan

**Affiliations:** 1 East China Academy of Inventory and Planning of National Forestry and Grassland Administration, Hangzhou 310001, China; zhuam@caf.ac.cn; 2 Research Institute of Subtropical Forestry, Chinese Academy of Forestry, Hangzhou 311400, China; yfllyin@163.com; 3 Zhejiang Forestry-Fund Management Center, Hangzhou 310012, China; 13738037801@163.com; 4 Zhejiang Academy of Forestry, Hangzhou 310023, China; 5 Forestry Farm of Jiande, Hangzhou 310023, China; 18069810771@163.com

**Keywords:** Moso bamboo, *Liquidambar formosana*, broadleaf transformation, soil nutrients, soil microbial structure and function

## Abstract

This study investigated how Moso bamboo (*Phyllostachys edulis*)–broadleaf mixed forests influence soil properties and microbial communities to support ecological function and sustainable bamboo forest management. Three forest types were examined: pure Moso bamboo stands (MB) and mixed stands with *Liquidambar formosana* (LB) or *Phoebe chekiangensis* (PB). Soil chemical properties, microbial diversity, and community composition were assessed using high-throughput sequencing, and functional taxa were correlated with soil nutrients. The results showed that mixed forests significantly influenced soil chemical properties. PB showed the lowest pH and highest total nitrogen (TN), while MB exhibited the highest soil organic matter (SOM) and total potassium (TK). LB maintained moderate TN, high SOM and TK, and stable pH, indicating a balanced nutrient profile. Although α-diversity did not differ significantly, β-diversity analysis revealed distinct microbial community structure (*p* < 0.01). LB was enriched with carbon-decomposing taxa (*Terriglobales* and *Sphingomonas*), PB with acid-tolerant, nitrogen-cycling groups (*Candidatus Binatus*), and MB with nitrogen-fixing taxa (*Nitrobacteraceae* and *Bradyrhizobium*). Co-occurrence network and functional pathway analyses indicated group-specific microbial associations and greater metabolic diversity in LB and PB. In conclusion, mixed Moso bamboo with broadleaf species significantly modified soil chemical properties and microbial community structure, with the Moso bamboo—*L. formosana* combination showing potential for improving soil nutrient status and microbial function.

## 1. Introduction

Mixed-species forest management is recognized as an effective approach to achieving sustainable forest development by enhancing soil properties and ecosystem functions [1]. The inclusion of broadleaf species improves soil quality through nutrient-rich and easily decomposable litter, which increases organic matter input, enhances soil structure, and improves water and nutrient retention [2]. Additionally, the chemical diversity of mixed-species litter and species-specific root exudates creates heterogeneous microenvironments that support diverse and functionally rich microbial communities [3]. These shifts in microbial composition promote key biogeochemical processes, such as organic matter decomposition and nutrient cycling, thereby enhancing soil ecosystem stability and resilience [4]. Collectively, mixed-species management contributes to more efficient and stable soil ecological functions, offering a promising strategy for long-term forest sustainability.

Moso bamboo (*Phyllostachys edulis*), a fast-growing and economically important species in southern China [5], has suffered from ecological degradation under long-term monoculture, including nutrient depletion, biodiversity loss, and reduced ecosystem resilience [6]. In response, the “broadleaf integration” strategy has been proposed, wherein ecologically compatible broadleaf species are introduced to establish mixed bamboo–broadleaf forests [7]. Previous studies have demonstrated that such integration enhances bamboo productivity, improves the microenvironment, and strengthens ecological functions [8]. However, while research has addressed bamboo-driven changes in plant or soil communities and nutrient dynamics [9,10,11,12], the mechanisms by which mixed-species management improves soil quality—particularly through microbial community responses and nutrient transformation processes—remain poorly understood.

Among candidate species for broadleaf integration, *Liquidambar formosana* Hance and *Phoebe chekiangensis* C. B. Shang are two ecologically adaptable native broadleaf trees with high ecological and economic value [13,14,15,16]. *L. formosana*, a deciduous pioneer, produces easily decomposable litter that accelerates early-stage organic matter accumulation and improves soil conditions [17]. *P. chekiangensis*, a valuable evergreen species with a well-developed root system, contributes to soil stabilization and long-term ecological benefits [15,16]. Their root architectures and resource strategies complement those of Moso bamboo, potentially reducing belowground competition and improving resilience to biotic and abiotic stressors. However, how these species influence soil microbial communities and nutrient dynamics in mixed forests remains largely unexplored.

This study investigates Moso bamboo mixed forests with *L. formosana* and *P. chekiangensis*, focusing on their effects on soil microbial diversity, community structure, and functional composition. By analyzing key microbial taxa and their associations with soil nutrients, this work aims to clarify the mechanisms by which mixed-species composition regulates soil quality. The findings will contribute to the scientific basis for ecological restoration and sustainable management of Moso bamboo forests.

## 2. Study Site

The study was conducted in the Lvhetang forest region of Shouchang Forest Farm, Jiande City, Zhejiang Province, China (29°25′45″–29°27′01″ N, 119°08′45″–119°11′00″ E), located within the Qianligang Mountain Range. The area features low mountains and hills, with elevations ranging from 200 to 804 m and slopes between 25° and 45°. It has a subtropical monsoon climate, with an average annual temperature of 17.6 °C (−13.9 °C to 42.2 °C), annual precipitation of approximately 1700 mm (mainly from April to June), relative humidity of 82%, and a frost-free period of 265 days.

The forest covers approximately 450 ha, including 370 ha of natural evergreen broadleaf forest, with over 300 woody plant species from 30 families. The dominant soil type is acidic yellow soil, 30–80 cm deep, with patches of eroded red soil. Moso bamboo plantations were established in 2007, covering over 600 acres, of which more than 300 acres have been converted into mixed bamboo–broadleaf forests using native broadleaf species.

## 3. Results

### 3.1. Effects of Moso Bamboo–Broadleaf Mixed Forests on Soil Chemical Properties

**Figure 1:**
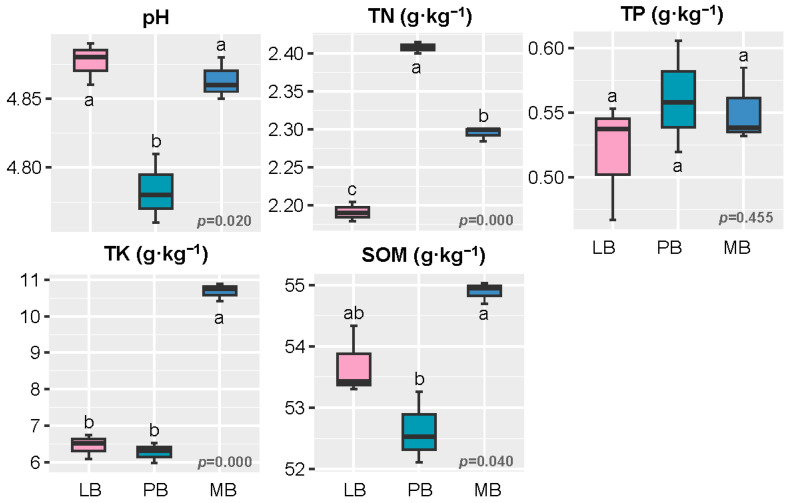
Variation in Soil Chemical Properties Across Different Forest Types. Note: Different lowercase letters indicate significant differences among forest types (*p* < 0.05). TN: total nitrogen, TP: total phosphorus, TK: total potassium, SOM: soil organic matter. LB represents the mixed forest of Moso bamboo and Liquidambar formosana, PB represents the mixed forest of Moso bamboo and Phoebe chekiangensis, and MB represents the pure Moso bamboo forest.

Figure 1 shows that the Moso bamboo–broadleaf mixed forest significantly influenced soil chemical properties (*p* < 0.05). Compared to the MB and LB groups, the soil pH in the PB group was significantly lower (*p* < 0.05), while TN content was significantly higher, with the lowest TN content observed in the LB group. TK and SOM contents in the MB group were significantly higher than those in the LB and PB groups (*p* < 0.05), with the lowest TK and SOM contents found in the PB group. No significant difference in TP content was observed among the three forest types.

### 3.2. Effects of Moso Bamboo–Broadleaf Mixed Forests on Soil Microbial Community Structure and Diversity

Illumina high-throughput sequencing of nine soil samples yielded a total of 135.41 Gbp of raw data. After strict quality control, 134.35 Gbp of high-quality data were retained. On average, each sample yielded approximately 14.92 Gbp, corresponding to about 50.15 million (50.15 M) reads per sample. The sequencing data had a Q20 value of 98.39% and a GC content of 60.18%, indicating high sequencing quality (Table 1). The data were then assembled using MEGAHIT software (v1.2.9), and Contigs longer than 500 bp were selected. This process resulted in 853,381 Scaftigs, with an average length of 783 bp, an N50 of 744 bp, and the longest fragment measuring 56,950 bp (Table 2).

**Figure 2:**
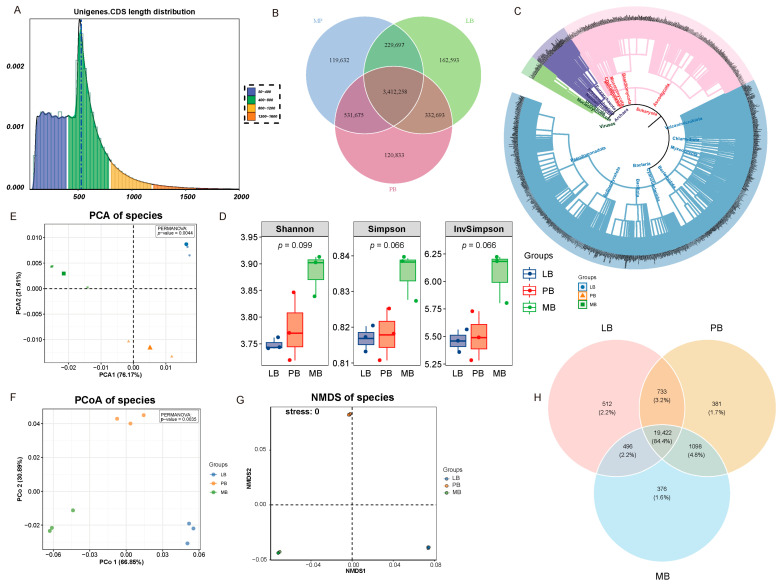
Functional Characteristics and Diversity Distribution of Soil Microbial Communities Across Different Forest Types. Note: (**A**) Length distribution of assembled coding sequences (CDS). (**B**) Distribution of non-redundant gene counts (Venn diagram). (**C**) Species relationship diagram identifying microbial genera at the genus level. (**D**) α-diversity of microbial communities (Shannon, Simpson, and Invsimpson indices). In the box plot, the horizontal line represents the mean, and the upper and lower boxes represent the upper and lower quartiles. (**E**–**G**) Principal component analysis (PCA), principal coordinate analysis (PCoA), and non-metric multidimensional scaling analysis (NMDS) plots. Different colors represent soil samples from different forest types. In the PCA and PCoA plots, the distance between sample points reflects their similarity in microbial community composition and relative abundance. Points that are closer together indicate similar community structures, whereas points that are farther apart suggest greater differences. In the NMDS plot, stress < 0.05 indicates an excellent fit, while 0.05 ≤ stress < 0.1 indicates a very good fit. (**H**) Venn diagram of the number of identified species at the taxonomic level among different groups. LB represents the mixed forest of Moso bamboo and Liquidambar formosana, PB represents the mixed forest of Moso bamboo and Phoebe chekiangensis, and MB represents the pure Moso bamboo forest.

Open reading frames (ORFs) for Scaftigs ≥500 bp were predicted using MetaGeneMark, and redundancy was removed using CD-HIT software (v4.5.8). A total gene length of 3039.26 Mbp was obtained, with an average gene length of 507.28 bp and a GC content of 60.83% (Figure 2A). In total, 3,412,258 non-redundant genes were annotated across the three sample groups (Figure 2B). Species annotation revealed that the Bacteria domain contained 21,146 species, Eukaryota had 1129 species, Archaea included 563 species, and Viruses contained 180 species. The Bacteria domain was the most diverse, encompassing 153 phyla, 135 classes, 294 orders, 716 families, and 2977 genera (Figure 2C).

Alpha diversity analysis (Shannon, Simpson, and Invsimpson indices) showed no significant differences in microbial community diversity among the treatment groups (*p* > 0.01, Figure 2D). However, beta diversity analysis revealed significant differences in microbial community structure based on phylogenetic distance. PCA, PCoA, and NMDS analyses clearly distinguished the three groups, with PERMANOVA tests all yielding significant results (*p* < 0.01). The NMDS analysis showed a good fit (Stress < 0.05) (Figure 2E–G). Venn diagram analysis further identified 19,422 shared mid-level species across the three groups (Figure 2H).

### 3.3. Effects of Moso Bamboo–Broadleaf Mixed Forests on Soil Microbial Community Structure

Analysis at the phylum and genus levels revealed significant impacts of different forest types on soil microbial community composition. At the phylum level, 17 major bacterial phyla were identified, including Acidobacteriota, Pseudomonadota, Actinomycetota, Verrucomicrobiota, Chloroflexota, Planctomycetota, Candidatus Binatota, Candidatus Rokuibacteriota, Myxococcota, Gemmatimonadota, Cyanobacteriota, Bacteroidota, Nitrospirota, Bacillota, Vulcanimicrobiota, Thermodesulfobacteriota, and Armatimonadota. Fungal phyla included Basidiomycota and Ascomycota, while Archaea was represented by Euryarchaeota.

**Figure 3:**
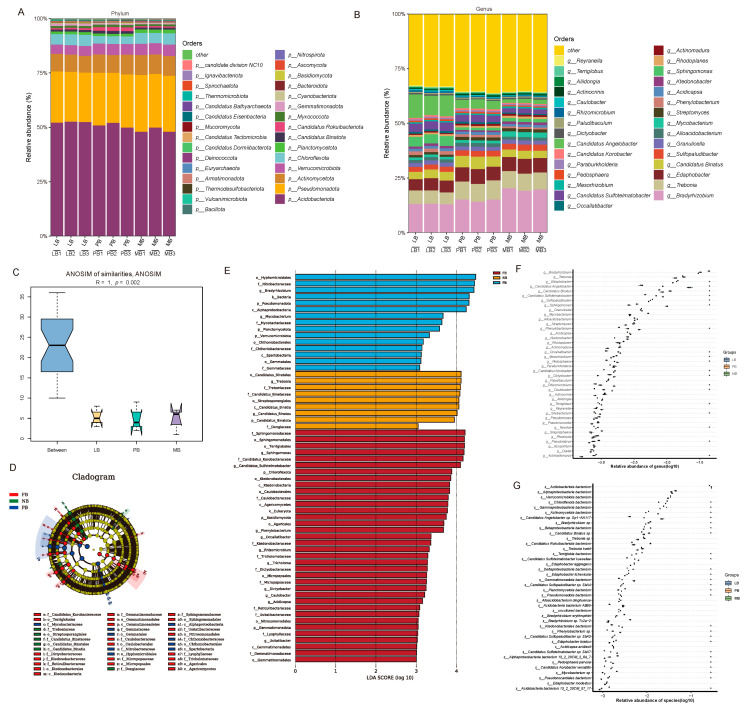
Soil Microbial Community Structure and Differential Species Identification Across Different Forest Types. Note: (**A**,**B**) Bar plots showing microbial composition at the phylum (**A**) and genus (**B**) levels, with different colors representing various taxonomic groups. (**C**) Non-parametric test (ANOSIM) based on the distance matrix. (**D**,**E**) Linear discriminant analysis effect size (LEfSe) with a selection criterion of LDA score > 3. Taxonomic labels follow the format: *p* = phylum, *c* = class, *o* = order, *f* = family, *g* = genus. (**F**,**G**) Kruskal–Wallis test for differential abundance of the top 40 most abundant microbes at the genus and species levels. LB represents the mixed forest of Moso bamboo and *Liquidambar formosana*, PB represents the mixed forest of Moso bamboo and *Phoebe chekiangensis*, and MB represents the pure Moso bamboo forest.

At the genus level, the top 30 most abundant microorganisms were primarily bacteria, predominantly from the phyla Acidobacteriota, Pseudomonadota, and Actinomycetota. Representative genera included *Bradyrhizobium*, *Trebonia*, *Edaphobacter*, *Candidatus Binatus*, *Sulfopaludibacter*, *Granulicella*, *Alloacidobacterium*, *Mycobacterium*, *Streptomyces*, *Phenylobacterium*, *Acidicapsa, Ktedonobacter*, *Sphingomonas*, *Rhodoplanes*, *Actinomadura*, *Occallatibacter*, *Candidatus Sulfotelmatobacter*, *Mesorhizobium*, *Pedosphaera*, *Paraburkholderia*, *Candidatus Korobacter*, *Candidatus Angelobacter*, *Dictyobacter*, *Paludibaculum*, *Rhizomicrobium*, *Caulobacter*, *Actinocrinis*, *Aliidongia*, *Terriglobus*, and *Reyranella* (Figure 3A,B).

ANOSIM similarity analysis indicated significant differences in microbial community composition between forest types (ANOSIM: R = 1, *p* = 0.002) (Figure 3C). LDA Effect Size analysis (LDA > 3) further highlighted forest-type-specific microbial biomarkers (Figure 3D,E): in the LB group, the biomarkers included Terriglobales (order), *Sphingomonas* (genus), and Candidatus Korobacteraceae (family); in the PB group, biomarkers included Dongiaceae (family), *Candidatus Binatus* (genus), Treboniaceae (family), Streptosporangiales (order), and Candidatus Binatales (order); in the MB group, biomarkers included Hyphomicrobiales (order), Nitrobacteraceae (family), *Bradyrhizobium* (genus), Pseudomonadota (phylum), and Alphaproteobacteria (class).

Kruskal–Wallis tests further revealed that the five most significantly different genera at the genus level were *Bradyrhizobium*, *Edaphobacter*, *Candidatus Sulfotelmatobacter*, *Candidatus Binatus*, and *Candidatus Angelobacter*, all of which are bacteria. At the species level, the top five significantly different species were *Chloroflexota bacterium*, *Actinomycetota bacterium*, *Candidatus Angelobacter* sp. Gp1−AA117, and *Bradyrhizobium* sp., all of which are also bacteria (Figure 3F,G).

### 3.4. Effects of Moso Bamboo–Broadleaf Mixed Forests on Soil Microbial Functional Structure and Its Coupling Relationship with Soil Nutrients

**Figure 4:**
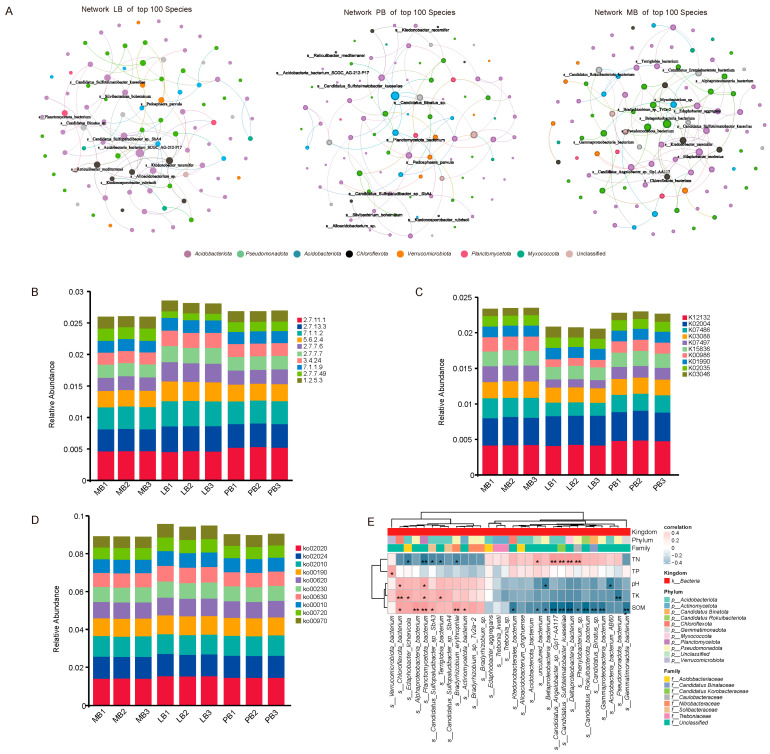
Analysis of Differential Microbial Functions and Their Correlation with Soil Nutrients Across Different Forest Types. Note: (**A**) Co-occurrence network of the top 100 microbial species. (**B**) Stacked bar chart showing the distribution of the top 10 enzyme commission (EC) functions. (**C**) Stacked bar chart showing the distribution of the top 10 KEGG Orthology (KO) functions. (**D**) Stacked bar chart showing the distribution of the top 10 KEGG pathway functions. (**E**) Correlation analysis between differential microbial species and soil nutrient contents. * indicates *p* < 0.05 (significant), ** indicates *p* < 0.01 (highly significant), and *** indicates *p* < 0.001 (extremely significant).

Co-occurrence network analysis of the top 100 species by abundance in each group revealed distinct microbial network structures. The LB group network consisted of 69 nodes and 81 edges, with the five species having the highest degree being *Acidobacteriota bacterium* SCGC AG-212-P17, *Ktedonobacter racemifer*, *Candidatus Sulfopaludibacter* sp. SbA4, *Candidatus Sulfotelmatobacter kueseliae*, and *Alloacidobacterium* sp. The PB group network comprised 63 nodes and 76 edges, with the top five species in terms of degree being *Actinomycetota bacterium*, *uncultured bacterium*, *Edaphobacter bradus*, *Candidatus Binatus* sp., and *Trebonia kvetii*. The MB group network included 67 nodes and 77 edges, with the highest degree species being Betaproteobacteria bacterium, *Edaphobacter modestus*, *Pseudomonadota bacterium*, *Candidatus Eremiobacterota bacterium*, and *Gammaproteobacteria bacterium* (Figure 4A).

Functional annotation analysis revealed the top 10 most abundant enzymes (EC numbers): EC.2.7.11.1, EC.2.7.13.3, EC.7.1.1.2, EC.5.6.2.4, EC.2.7.7.6, EC.2.7.7.7, EC.3.4.24, EC.7.1.1.9, EC.2.7.7.49, and EC.1.2.5.3. The top 10 most abundant KEGG Orthology (KO) annotations included K12132, K02004, K07486, K03088, K07497, K15836, K00986, K01990, K02035, and K03046. The most enriched metabolic pathways among the top 10 were ko02020 (Two-component system), ko02024 (Quorum sensing), ko02010 (ABC transporters), ko00190 (Oxidative phosphorylation), ko00620 (Pyruvate metabolism), ko00230 (Purine metabolism), ko00630 (Glyoxylate and dicarboxylate metabolism), ko00010 (Glycolysis/Gluconeogenesis), ko00720 (Carbon fixation pathways in prokaryotes), and ko00970 (Aminoacyl-tRNA biosynthesis) (Figure 4B–D).

Further analysis of the correlation between the top 30 core microbial species and soil nutrients revealed that *Verrucomicrobiota bacterium*, *Chloroflexota bacterium*, *Edaphobacter lichenicola*, *Alphaproteobacteria bacterium*, *Planctomycetota bacterium*, *Candidatus Sulfopaludibacter* sp. SbA3, *Terriglobia bacterium*, *Bradyrhizobium erythrophlei*, *Actinomycetota bacterium*, and *Bradyrhizobium* sp. Tv2a−2 were significantly positively correlated with SOM and TK (*p* < 0.05), but negatively correlated with TN. Conversely, *Betaproteobacteria bacterium*, *Candidatus Angelobacter* sp. Gp1-AA117, *Candidatus Sulfotelmatobacter kueseliae*, *Deltaproteobacteria bacterium*, *Phenylobacterium* sp., *Candidatus Rokuibacteriota bacterium*, *Candidatus Binatus* sp., *Gammaproteobacteria bacterium*, and *Gemmatimonadota bacterium* showed significant negative correlations with SOM and significant positive correlations with TN (Figure 4E).

## 4. Discussion

The mixed planting of Moso bamboo with different broadleaf tree species significantly influenced soil chemical properties. Compared with the MB and LB groups, the PB group exhibited pronounced soil acidification, as indicated by a significantly lower pH, which may be attributed to the release of organic acids during the decomposition of *P. chekiangensis* litter [15,16]. Additionally, the PB stand had the highest TN content, whereas the LB group had the lowest, suggesting that different companion tree species regulate soil nitrogen accumulation through mechanisms such as litter quality and rhizosphere nitrogen transformation [15,18]. The MB group showed significantly higher levels of TK and SOM than the mixed stands, implying that bamboo monocultures may be more conducive to potassium and organic matter accumulation [19]. The LB group followed closely in TK and SOM levels, indicating that mixing Moso bamboo with *L. formosana* may offer advantages in improving soil fertility. No significant differences in TP were observed among the three forest types, possibly due to the low mobility of phosphorus in soil and its limited short-term response to vegetation changes [20,21]. Overall, the type of broadleaf species mixed with Moso bamboo plays a key role in shaping soil nutrient dynamics, with the Moso bamboo–*L. formosana* combination showing greater ecological potential for maintaining soil fertility.

The mixed planting of Moso bamboo with different broadleaf species did not significantly alter soil microbial diversity but substantially reshaped the structure and composition of the microbial community, indicating that forest type strongly influences microbial assembly processes and ecological functions. Although bacteria remained the dominant taxa across all three forest types—primarily from the phyla Acidobacteriota, Pseudomonadota, and Actinomycetota—the distribution of dominant genera varied notably among stands, suggesting that tree species composition exerts a strong selective effect on microbial community structure [22,23]. In the LB group, taxa such as *Terriglobales* and *Sphingomonas*, known for their roles in organic matter decomposition and carbon mineralization [24,25], were enriched, potentially enhancing soil carbon cycling and facilitating adaptation to the heterogeneous environment of mixed forests. In the PB group, enriched taxa like *Candidatus Binatus* and *Treboniaceae* were associated with carbon transformation and nitrification under acidic conditions [26,27], implying the activation of specific carbon and nitrogen metabolic pathways. By contrast, the MB group was dominated by classic nitrogen-fixing and nitrifying bacteria such as *Nitrobacteraceae* and *Bradyrhizobium*, indicating a higher potential for nitrogen fixation and transformation [28,29], which may help maintain nitrogen supply within the system. Additionally, taxa such as *Candidatus Sulfotelmatobacter* and *Candidatus Angelobacter* were broadly distributed in the mixed forests, potentially participating in key processes such as heterotrophic denitrification and organic nitrogen mineralization [30,31], highlighting the regulatory role of mixed forest structure in shaping nitrogen cycling pathways. Significant differences at the genus and species levels—such as shifts in the abundance of *Bradyrhizobium* and *Chloroflexota bacterium*—further underscore the selective influence of vegetation composition on key functional microbial groups in soil ecosystems.

The conversion of Moso bamboo monocultures through broadleaf integration significantly altered the functional structure of soil microbial communities and their coupling with soil nutrients [11,12,32]. Distinct differences in microbial co-occurrence network characteristics were observed among the mixed forest types, indicating that stand composition influences the ecological interactions and functional potential of core microbial taxa [32]. In the LB group, species such as *Acidobacteriota bacterium* SCGC AG-212-P17, *Ktedonobacter racemifer*, and *Candidatus Sulfopaludibacter*—closely associated with organic matter decomposition and carbon mineralization [30,33,34]—were enriched, suggesting that this mixed forest enhances soil carbon cycling capacity. In the PB group, core species such as *Edaphobacter bradus* and *Candidatus Binatus sp.* were more active under acidic and nitrogen-rich conditions [30], highlighting their key roles in nitrogen transformation. In contrast, the MB group was dominated by taxa such as *Betaproteobacteria bacterium* and *Gammaproteobacteria bacterium*, which exhibit strong nitrogen mineralization potential [35,36], indicating selective enrichment of specific nitrogen-cycling microbes under monoculture conditions.

Functional annotation revealed that microbial communities across forest types exhibited differences in signaling pathways, energy metabolism, and carbon and nitrogen metabolic processes, reflecting microbial adjustments in functional redundancy and metabolic strategies under different soil environments [37,38]. Notably, enriched pathways such as carbon fixation (ko00720), glycolysis/gluconeogenesis (ko00010), and pyruvate metabolism (ko00620) pathways were more active in the LB and PB groups than in the MB group, indicating enhanced microbial involvement in soil carbon cycling under mixed stands. The upregulation of glycolysis and pyruvate metabolism may promote energy production and organic matter decomposition, while increased carbon fixation suggests greater microbial autotrophic activity and carbon assimilation [33,39]. Although nitrogen metabolism was not among the top enriched pathways, the higher abundance of nitrogen-transforming taxa in the PB and MB groups implies active nitrogen cycling processes such as ammonification and nitrification.

These functional differences imply that microbial communities in mixed forests may play a more dynamic role in sustaining soil fertility. Correlations between microbial taxa and soil nutrients further confirmed functional differentiation. In the LB group, microbes significantly positively correlated with SOM and TK—such as *Bradyrhizobium erythrophlei* and *Chloroflexota bacterium*—were primarily involved in carbon cycling and potassium release [33], potentially enhancing SOM accumulation and potassium availability. In contrast, microbes enriched in the PB and MB groups, including *Candidatus Binatus* and *Gammaproteobacteria bacterium*, were positively correlated with TN but negatively with SOM, suggesting that these taxa may contribute to increased organic matter consumption by promoting nitrogen transformation [30]. Although soil enzyme activity was not directly measured in this study, the observed functional profiles align with microbial capacities for key enzymatic processes (e.g., nitrate reductase, urease, cellulase), indicating potential feedbacks to nutrient turnover and ecosystem nutrient-use efficiency. Future studies integrating metagenomic prediction with soil enzyme assays could further validate the microbial contributions to nutrient cycling under different mixed forest compositions.

## 5. Materials and Methods

### 5.1. Experimental Design

Since 2015, a broadleaf conversion experiment has been conducted in a Moso bamboo forest, with 1-year-old seedlings of *L. formosana* (LB) and *P. chekiangensis* (PB) interplanted beneath the bamboo canopy. The seedlings, with an initial height of 0.7 m and base diameter of 0.7 cm, were planted in rows at a 4 m × 4 m spacing. Prior to planting, bamboo stems older than four years were thinned to a density of 1000 stems per hectare. Maintenance activities, including the removal of bamboo over four years old and the clearing of underbrush, were performed every three years. Dead plants were replaced throughout the experimental period. In March 2025, experimental samples were collected, with forest stand growth details provided in Supplementary Table S1.

Three 20 m × 20 m plots were established for each forest type, totaling nine plots. Soil samples were collected from the 0–20 cm layer using a five-point sampling method. Samples from each plot were pooled into a composite sample, with three replicates for each forest type. The mixed samples were divided into three portions: one transported on dry ice and stored at −80 °C for soil metagenomic sequencing; one air-dried indoors at room temperature for chemical analysis; and one sieved through a 0.15 mm mesh for measurements of total nitrogen, phosphorus, potassium, and organic matter, with the remaining sieved through a 2 mm mesh for pH determination. All analyses were conducted in triplicate.

### 5.2. Soil DNA Extraction

Soil samples were collected in sampling tubes and immediately stored at −80 °C until processing. The abundance of soil microorganisms was analyzed using metagenomic sequencing. DNA was extracted from the soil samples using the E.Z.N.A™ Mag-Bind Soil DNA Kit (Omega Bio-Tek, Norcross, GA, USA). Amplified products were sequenced on the Illumina MiSeq platform (Illumina, San Diego, CA, USA), with DNA libraries constructed according to standard protocols. The libraries were generated using the NEBNext Ultra DNA Library Prep Kit for Illumina (New England Biolabs, Ipswich, MA, USA) and assessed for quality using the Agilent 2100 Bioanalyzer (Agilent, Santa Clara, CA, USA), with quantification performed via real-time PCR. Whole-genome sequencing was conducted on the NovaSeq 6000 system (Illumina), with all samples sequenced using paired-end reads of 150 base pairs (bp). The target dataset size was 15 Gb.

### 5.3. Metagenomic Sequencing and Data Analysis

Raw sequencing data were processed to obtain high-quality reads. First, Cutadapt v1.9 was used to remove sequencing adapters, followed by quality trimming of low-quality reads using fqtrim v0.94 with a sliding window algorithm. The filtered reads were assembled de novo using MEGAHIT to construct metagenomes for each sample. Coding sequences (CDSs) were predicted from the assembled contigs using MetaGeneMark v3.26. The resulting CDSs were clustered using CD-HIT v4.6.1 to obtain unigenes. The abundance of unigenes in each sample was estimated based on the number of aligned reads using Bowtie2 v2.2.0 and expressed as TPM (Transcripts Per Million). Functional and taxonomic annotations were conducted by aligning unigenes against the NCBI NR database using DIAMOND v0.9.14, including GO, KEGG, eggNOG, CAZy, CARD, PHI, MGEs, and VFDB databases.

Alpha diversity indices (Shannon, Simpson, and Inverse Simpson) were calculated using the “vegan” package in R, and statistical differences between groups were evaluated using Kruskal–Wallis non-parametric tests, followed by Dunn’s post hoc pairwise comparisons to determine significance levels between specific groups. Beta diversity was assessed via principal component analysis (PCA) and principal coordinates analysis (PCoA) based on the Bray–Curtis metrics, both performed with the “vegan” package in R. Statistical differences in community composition between groups were evaluated using permutational multivariate analysis of variance (PERMANOVA) [40]. Non-metric multidimensional scaling (NMDS) based on Bray–Curtis dissimilarity matrices was used to visualize group differences, with stress values used to assess goodness-of-fit. Analysis of similarity (ANOSIM), a non-parametric test based on distance matrices, was performed to assess microbial community dissimilarities among groups.

Bar plots of taxonomic composition were generated using the R package ggplot2 (v3.5.1), and differential taxa were identified using linear discriminant analysis effect size (LEfSe), with an LDA score threshold of >3 [41,42]. The top 40 genera and species were analyzed using the Kruskal–Wallis test. Microbial co-occurrence networks were constructed using SparCC3 (https://github.com/JCSzamosi/SparCC3 (accessed on 1 April 2025)), with correlation thresholds set at |r| > 0.25 and *p* < 0.015 [43,44]. Keystone taxa (top 1% by node degree) were identified, and networks were visualized in Gephi (v0.9.2) using the Fruchterman–Reingold layout [45]. Additionally, correlations between microbial taxa and soil physicochemical properties were assessed using the corrplot package in R (v0.95) based on Spearman’s rank correlation analysis.

### 5.4. Soil Nutrient Analysis

Soil pH was measured using a pH meter at a soil-to-water ratio of 1:2.5. Total nitrogen (TN) and total carbon (TC) contents were determined using an automated carbon-nitrogen analyzer (Vario Max, Elementar Analysensysteme GmbH, Langenselbold, Germany). Total potassium (TK) was measured with an atomic absorption spectrophotometer (AA-7003, Beijing Sanxiong Technology Co., Ltd., Beijing, China) after digestion with a nitric-perchloric acid mixture. Soil organic matter (SOM) was determined via potassium dichromate oxidation and quantified using a UV–visible spectrophotometer (TU-1901, Beijing Purkinje General Instrument Co., Ltd., Beijing, China). All data are presented as mean ± standard deviation (SD). One-way analysis of variance (ANOVA) was performed to assess differences among groups, followed by Tukey’s post hoc test to determine pairwise significance at *p* < 0.05. Graphs were generated using GraphPad Prism (v8.0, GraphPad Software, Boston, MA, USA).

## 6. Conclusions

The mixed planting of Moso bamboo with broadleaf tree species significantly reshaped the structure and functional expression of soil microbial communities, thereby regulating the mechanisms driving soil nutrient cycling. Among the mixtures, the combination of Moso bamboo and *L. formosana* exhibited the most favorable outcomes, including enhanced carbon cycling potential, sustained soil fertility, and optimized microbial functional structure, indicating strong ecological regulatory capacity. It is therefore recommended that, during the expansion or restoration of Moso bamboo forests, broadleaf species such as *L. formosana*—which possess strong ecological adaptability and produce high-quality litter—be prioritized for mixed planting. This approach would promote the restoration of soil ecological functions and support sustainable forest management and quality improvement of bamboo ecosystems. As the system remains in a stage of ecological succession, long-term monitoring of soil and microbial dynamics is essential to track ecosystem development and functional stabilization.

## Figures and Tables

**Table 1:** Statistics of Metagenomic Sequencing Data for Soil Samples from Different Forest Types.

Sample ID	Raw_Base (G)	Clean_Base (G)	Clean_Q20 (%)	Clean_Q30 (%)	Clean_GC (%)	Effective (%)
MB1	15.36	15.26	98.48	96.06	60.19	99.36
MB2	14.63	14.53	98.44	95.98	60.23	99.29
MB3	16.91	16.81	98.5	96.04	60.19	99.37
LB1	10.38	10.07	97.82	94.33	60.38	97.02
LB2	13.97	13.9	98.44	95.94	60.01	99.46
LB3	17.38	17.27	98.44	95.97	60.22	99.39
PB1	15.96	15.89	98.5	96.09	60.18	99.56
PB2	15.31	15.23	98.5	96.06	60.14	99.53
PB3	15.51	15.39	98.46	95.87	60.11	99.18

**Table 2:** Statistics of Metagenomic Assembly Data for Soil Samples from Different Forest Types.

Sample ID	Total Length (bp)	Scaftigs Num	Average Length (bp)	N50 Length (bp)	N90 Length (bp)	Max Length (bp)
MB1	730,229,717	899,185	812.1	772	533	81,211
MB2	675,247,105	835,002	808.68	768	533	105,128
MB3	831,806,140	1,014,565	819.86	781	534	89,391
LB1	324,951,331	456,067	712.51	676	524	18,717
LB2	512,751,453	697,120	735.53	699	526	21,800
LB3	714,921,510	945,420	756.19	719	528	26,918
PB1	795,079,007	993,465	800.31	761	533	46,135
PB2	757,034,068	940,087	805.28	765	533	64,636
PB3	717,743,789	899,519	797.92	757	533	58,615

## Data Availability

Data are contained within the article and Supplementary Materials.

## Supplementary Materials

The following supporting information can be downloaded at: https://www.mdpi.com/article/10.3390/plants14121868/s1, Table S1: Growth Characteristics of the Experimental Forests.
