# Supplementary material for: Microbial-Mediated Soil Nutrient Enhancement in Moso Bamboo–Liquidambar formosana vs. Phoebe chekiangensis Mixed Plantings
Source: Plants (Basel). 2025 Jun 18;14(12):1868. doi: 10.3390/plants14121868 (PMC12196586; doi:10.3390/plants14121868)
Supplement: Supplementary file 1 [file plants-14-01868-s001.zip › plants-3659365-supplementary.pdf]

Table S1. Growth Characteristics of the Experimental Forests

Groups	Moso bamboo			*Liquidambar formosana*			*Phoebe chekiangensis*			Altitude /m	Slope /°	Aspect
	Density /(Individual·hm^{-2})	Average breast diameter /cm	Average height /cm	Density /(Individual·hm^{-2})	Average breast diameter /cm	Average height /cm	Density /(Individual·hm^{-2})	Average breast diameter /cm	Average height /cm			
MB	2,750	4.28±1.81	15.78±1.36	-	-	-	-	-	-	261	21	Southeast
LB	1,250	4.02±1.18	15.12±1.74	517	9.11±1.69	8.86±1.75	-	-	-	263	21.3	Southeast
PB	1,000	5.50±1.04	16.49±1.12	-	-	-	460	7.28±1.03	5.79±1.46	259	20.7	Southeast
